# Book Reviews

**Published:** 1873-06-15

**Authors:** 


### Book Reviews.

Medical and Surgical History of the War of the Rebellion. Part I., Vols. I and II. War Department Surgeon-General'sOffice, Washington, D. C.

These two large volumes, comprising nearly 2,000 pages, give an historical record of over six and a half-million of medical and surgical cases that occurred during the four years of war. Of these cases, 304,369 terminated fatally. No similar work of equal magnitude has ever been undertaken by any government. The French and English governments each published some incomplete statistics of the military medicine and surgery of the Crimean war. The American government, however, took measures, early in the progress of the war, to collect the statistics on a more complete and extended scale.

The pension law requiring each pensioner to make at least an annual appearance before some government officer, has been of great assistance in keeping track of the more interesting cases, and enabling an accurate record of their history and progress to be kept.

For instance, the wanderings of private Patrick Hughes, formerly of Co. K, 4th N. Y. volunteers, who still continues to live after the perforation of his cranium by a conical musket-ball, has been diligently followed up for nine years, It is a remarkable case of a continuation of life and work under most adverse circumstances, The ball entering near the inner posterior angle of the right parietal, emerged at a higher point of the left parietal, and after traversing the brain made a large exit wound. A piece of the skull was carried away of the size of six square inches, and through the aperture thus formed the pulsation of the arteries of the brain could be plainly seen. Yet this man livde to leave the army, and is strong enough to be a hard-working puddler in an iron-foundry. Fourteen other cases occurred during the war, where soldiers received gunshot perforations of the cranium, and lived; in fifty-four more cases the patients died after they were carried to the hospital.

In the medical volume we find the established history of 19,971 deaths from pneumonia, one of the most prevalent of all causes of loss of life among the troops in hospital, and only exceeded by the several forms of diarrhœa and dysentery, which carried off 44,558 soldiers by death, and sent home 17,748 more under their discharges. Over 20,000 were dismissed on account of incipient tuberculosis, and 12,000 for rheumatism.

The statistics show conclusively that the average recovery of the sick and wounded was larger in open tents than in hospitals, The open, free ventilation of the one was more than an equivalent for the superior accommodations of the other.

It is to be regretted that the limited appropriation from Congress enabled but a small edition of these valuable works to be issued. There is a movement now on foot to petition Congress for an appropriation to enable another and larger edition to be issued for general distribution to the profession.

These volumes were prepared under the direction of Surgeon-General Jas. K. Barnes, the medical record being drawn up by Dr. J. J. Woodward, U. S. A., and the surgical by Dr. Geo. A. Otis.

Especial credit is due to these gentlemen for the careful and thorough manner in which this vast fund of valuable matter has been gathered together.

The Mechanism of the Vesicles of the Ear and Membrana Tympani. By H. Helmholtz. Translated from the German, with the author's permission, by Albert H. Buck and Norwood Smith, of New York. With twelve illustrations. Wm. Wood & Co., New York.

